# Maternal suicide – Register based study of all suicides occurring after delivery in Sweden 1974–2009

**DOI:** 10.1371/journal.pone.0190133

**Published:** 2018-01-05

**Authors:** Henrik Lysell, Marie Dahlin, Alexander Viktorin, Elsa Ljungberg, Brian M. D'Onofrio, Paul Dickman, Bo Runeson

**Affiliations:** 1 Department of Clinical Neuroscience, Karolinska Institutet, Centre for psychiatry research, Stockholm, Sweden; 2 Department of Medical Epidemiology and Biostatistics, Karolinska Institutet, Stockholm, Sweden; 3 Department of Hematology, Oncology and Radiation Physics, Skåne University Hospital, Lund, Sweden; 4 Department of Psychological and Brain Sciences, Indiana University, Bloomington, IN, United States of America; National Institute of Child Health and Human Development, UNITED STATES

## Abstract

**Background:**

Recent research suggests that having a newborn child is associated with substantially reduced risk for maternal suicide. We studied postpartum suicides in a national cohort of mothers and the role of mental disorder, self-harm and delivery related factors.

**Methods:**

We used a nested case-control design with data from Swedish registries. The cohort consisted of all women given birth in Sweden 1974–2009. Mothers who died by suicide during follow-up were considered cases (n = 1,786) and risk of suicide was estimated with proximity to delivery as the explanatory variable. In a second step, association between suicide during the first year following delivery (n = 145) and mental disorder, self-harm and delivery related variables risk factors were analyzed.

**Results:**

The first postpartum year was associated with a lower risk of suicide, compared to later (RR 0.80, 95%CI 0.66–0.96), which was unaltered after adjustment for socio-economic status and history of self-harm (aRR 0.82, 95%CI 0.68–0.99). Compared to living mothers, suicide victims of the postpartum year more often had affective disorders (aRR 133.94, 95%CI 45.93–390.61), psychotic disorders (aRR 83.69, 95%CI 36.99–189.31) and history of self-harm (aRR 47.56, 95%CI 18.24–124.02). The aRR of stillbirth was 2.66 (95%CI 0.63–11.30).

**Conclusions:**

We found only a weak negative association between childbirth during the preceding year and suicide, when using mothers as controls. A severe mental disorder after delivery and a history of self-harm was strongly associated with increased risk of suicide in the postpartum year and may inform the clinical assessment postpartum.

## Introduction

Pregnancy and recent delivery are considered to protect against suicide [1]. Previous studies have shown that pregnant women and women in the postpartum period are less likely to commit suicide compared to women in the general population [1–3]. Suicide rates six times lower than expected has been reported among mothers during the first year following childbirth [1]. In general, having children, and young children in particular, appears associated with lower rates of suicide [4] and it is still unknown whether the apparent protective effect of recent childbirth remains when comparison is made between newly delivered women and mothers in general.

The postpartum period is associated with increased risk of mental disorders [5]. These problems range in severity from the common postpartum blues and postpartum depression, which affects up to 20% of mothers, to the more rare postpartum psychosis, with a prevalence of 0.1% [6, 7]. Overall, mental disorders are strongly associated with suicide [8, 9]; thus, onset in the postpartum period is likely to increase the presumed low risk of suicide. In a Danish study, mothers with postpartum psychiatric admission had a 70-fold increased risk of suicide during the first year after delivery, compared to women in the general Danish population [10]. Further, in a fairly small US study, women with mental disorder and/or substance use disorder had an elevated risk of postpartum suicide attempt compared to healthy mothers [11].

Delivery related factors, such as acute caesarean section [12] and childcare stress [13], seem to increase the risk of postpartum depression, whereas having a stillborn child increases the risk of maternal suicide [4, 14].

Even though suicides after childbirth are not highly prevalent, preventive measures are of obvious importance with regard to its severe consequences, including late psychological consequences for the children [15]. In order to further elucidate factors associated with suicide in women in the postpartum period we set up two objectives for this study. First, we aimed to explore the association between recent delivery and suicide amongst mothers; specifically, whether the postpartum period is related to suicide risk when adjusting for socio-economic status and when analysis is made within a cohort of mothers and not women in general. Second, we explored the extent to which suicides occurring within the first year after delivery are associated with current mental disorder, history of self-harm and delivery related factors.

## Method

We used a nested case-control design, based on Swedish national registry data, including the Medical Birth Register that contains information on all births, including stillbirths, in Sweden from 1973 and onwards [16]. The Cause of Death Register, the National Patient Register, the Total Population Register, the National Census and the Education Register were all merged by the use of the personal identification number, given to every citizen at birth and to immigrants when obtaining permit of residence [17].

All women registered for having given birth in Sweden were considered at risk and included in the cohort at the time of delivery from January 1, 1974 to December 31, 2009. Mothers remained in the cohort until death, emigration or end of follow-up, December 31, 2009. The cohort consisted of a total of 1 848 941 mothers.

For our first aim, we used three separate time interval measures of recent delivery as exposures: within the first 42 days, according to the World Health Organization definition of maternal death [18, 19], within six months and within one year after delivery. Cases were identified within the cohort of mothers if they had a registration of death by suicide before the age of 40. Restriction on age was made, in order to avoid inclusion of suicides among post-menopausal mothers, who would not have the possibility of exposure to recent delivery; mean maternal age at delivery in Sweden was 26–30 years during the study period [20]. Suicides included both verdicts of certain and uncertain intent (ICD8/9: E950-E959, E980-E989, ICD-10: X60-X84, Y10-Y34) to avoid underestimation, consistent with previous studies [21, 22]. Matched controls, on maternal year of birth, were randomly selected within the cohort of mothers. Ten controls were assigned to each case.

For our second aim, we used current mental disorder, a history of self-harm and delivery related factors as exposures. A current mental disorder was defined as registered at discharge from hospital care during the year preceding index event. A history self-harm was recorded if there was any lifetime registration of self-harm at discharge from hospital care. Delivery related factors were recorded at the most recent delivery. Cases were mothers who died by suicide within one year after childbirth, regardless of age. Controls were randomly selected from mothers in the cohort without suicide during the first year after delivery and matched on maternal year of birth and on year and month of most recent delivery. Due to few cases and rare exposure variables, we aimed to match 100 controls to every case. The matching thus yielded a sample of cases and controls, respectively, who were equal in age and who had a child of the same age.

### Mental disorder and self-harm

The National Patient Register holds information on all psychiatric inpatient admissions in Sweden from 1973 and the register has showed fair to excellent coverage and validity of diagnoses [23]. We divided codes into four diagnostic categories: *psychotic disorders* (ICD-8: 291, 295, 297, 298, 299 ICD-9: 291–292, 295, 297, 298 and ICD-10: F20-F25, F28-F29, F32.3, x.5 in F10-F19), *affective disorders* (ICD-8: 296, 300.4, ICD-9: 296A-296E, 296W, 296X, 300E, 311, ICD-10: F30-F39 except 32.3), *personality disorders* (ICD-8: 301, ICD-9: 301 and ICD-10: F60) and *substance use disorders* included (ICD-8: 303–304, ICD-9: 303–304, 305A, 305X, ICD-10: F10-F19 except x.5). We further constructed the variable *any mental disorder*, which included all the above specified groups, and, in addition, phobic disorders, anxiety disorders, obsessive disorders, eating disorders and adjustment disorders (ICD-8: 300 except 300.4, ICD-9: 300 except 300E, 307B, 307F, ICD-10: F40-42, F44-F45, F48, F50). *History of self-harm* was defined as discharge under the verdict of certain and uncertain intent (ICD-8/9: E950-E959, E980-E989, ICD-10: X60-X84, Y10-Y34). Since the register lacks information on suicidal intent we used the term self-harm in line with previous research [24], capturing both suicidal attempts and non-suicidal self-harm.

### Maternity data and demography

Data on delivery by caesarean section, stillbirth and multiple birth (twins or more) were gathered from the Medical Birth Register. Previous deliveries, other than most recent, were registered in order to account for any effect of being a first time mother. No further sibling data were used in the analyses.

Educational level was obtained from the National Census and Education Register and dichotomized into high and low (completion of nine years compulsory school or less). Immigrant status was defined as born outside of Sweden.

### Statistical analysis

To estimate risk factors for suicide after delivery, conditional logistic regression was used. The Proc logistic command in SAS yielded rate ratios (RR) with 95% confidence intervals (CI) [25], taking into account the dependence between cases and controls due to matching.

For the first objective, crude estimates of the association of recent delivery and suicide were calculated and subsequently adjusted for immigrant status and low educational level.

For the second objective, we estimated associations between suicide within one year after childbirth and mental disorders first bivariate and secondly in an adjusted model containing all disorders, immigrant status and educational level. Associations between delivery related variables and suicide were analyzed first bivariate and then with immigrant status and educational level in an adjusted model.

All statistical analyses and the construction of the datasets were performed using SAS (SAS Institute, Cary, NC, USA) version 9.4.

The study had ethical approval by the Regional ethical review board in Stockholm (2009/939-31/5).

## Results

We identified 1,786 suicides among women aged 18–40 who had given birth 1974–2009. Among suicide cases, 141 mothers (7.9%) had delivered within one year before their death and 72 (4.0%) within six months before death (Table 1). Childbirth within the preceding year, was negatively associated with suicide (RR 0.81, 95% CI 0.68–0.97). After adjustment for educational level and immigrant status, the association was unaltered (aRR 0.82, 95% CI 0.68–0.99). Delivery within 6 months and ≤42 days before suicide, yielded statistically non-significant point estimates of aRR = 0.80 and aRR = 0.76 respectively.

**Table 1:** Association of recent delivery and suicide among mothers aged 18–40 in Sweden 1974–2009.

	Suicide N = 1,786		Controls N = 17,860		P Value	Crude Analysis		Adjusted Analysis^a^	
	n	(%)	n	(%)		RR^b^	CI 95%	RR^b^	CI 95%
Delivery within the last year	141	(7.9)	1,705	(9.9)	0.018	0.81	(0.68–0.96)	0.82	(0.68–0.99)
Delivery within the last six months	72	(4.0)	867	(5.0)	0.120	0.82	(0.64–1.05)	0.80	(0.62–1.03)
Delivery within the last 42 days	15	(0.8)	184	(1.1)	0.444	0.81	(0.43–1.24)	0.76	(0.44–1.30)

a) Adjusted for low educational level and immigrant status.

b) Rate ratios from logistic regression with matching on maternal year of birth.

In the sample of mothers with suicide during the first year after childbirth, regardless of age at suicide, we found 145 mothers (mean age: 30 years, range: 17.9–44.1 years). These mothers had more often a low educational level (55.9% vs. 18.2%, χ^2^ = 139.5, p<0.001) (Table 2). Current mental disorder and history of self-harm in the postpartum period were more common among cases than controls and the differences in proportions were all highly significant. Forty-four mothers (30.3%) had a current mental disorder. The most common diagnostic categories were psychotic disorders (12.4%) and affective disorders (11.7%). Thirty mothers (20.7%) had an admission due to self-harm occurring before the suicide (Table 2).

**Table 2:** Association of suicide during the first year after delivery and, educational and immigrant status, mental disorders and previous self-harm.

	Suicide N = 145		Controls N = 13,786		P Value	Crude Analysis		Adjusted Analysis^a^	
	n	(%)	n	(%)		RR^b^	CI 95%	RR^b^	CI 95%
Low educational level	81	(55.9)	2,412	(17.5)	<0.001	6.27	(4.45–8.84)	5.14	(3.45–7.64)
Born outside of Sweden	32	(22.1)	1,820	(13.2)	<0.01	1.82	(1.22–2.72)	1.20	(0.75–1.91)
*Mental disorders*^*c*^									
Any mental disorder^e^	41	(28.3)	41	(0.3)	<0.001	124.85	(76.03–205.00)	–	–
Psychotic disorders^e^	18	(12.4)	17	(0.1)	<0.001	110.90	(55.65–221.03)	83.69	(36.99–189.31)
Affective disorders^e^	17	(11.7)	10	(0.1)	<0.001	174.08	(77.02–393.49)	133.94	(45.93–390.61)
Personality disorders^e^	3	(2.1)	2	(0.0)	<0.01	149.70	(25.01–895.90)	2.42	(0.20–29.34)
Substance use disorders^e^	5	(3.4)	7	(0.1)	<0.001	61.62	(16.97–221.32)	16.87	(3.54–80.34)
History of self-harm^d^^,^^e^	30	(20.7)	155	(1.1)	<0.001	132.65	(63.29–278.01)	47.56	(18.24–124.02)

a) Adjusted for all other variables.

b) Rate ratios from logistic regression with matching on maternal year of birth.

c) Diagnoses at discharge ≤1 year prior to suicide/index date.

d) Registered in the National Patient Register before index event.

e) Reference category is absence of the variable.

The risk of suicide within one year after delivery was increased for all diagnostic categories. Most prominent was the association between affective disorder and suicide (RR 174.08, 95% CI 77.02–393.49). Adjustment for low educational level, immigration status, other mental disorder and self-harm attenuated the association (aRR 133.94, 95% CI 45.93–390.61). Self-harm constituted a strong independent risk factor for suicide in the first year after childbirth (aRR 47.56, 95% CI 18.24–124.02). Further, substance use disorder (aRR 16.87, 95% CI 3.54–80.34) and a low educational level (aRR 5.14, 95% CI 3.45–7.64) remained independent risk factors after adjustment (Table 2). A stillborn child was non-significantly associated with suicide (aRR 2.66, 95% CI 0.63–11.30) (Table 3).

**Table 3:** Association of suicide during the first year after delivery and delivery related factors.

	Suicide N = 145		Controls N = 13,786			Crude Analysis		Adjusted Analysis^a^	
	n	(%)	n	%	P Value	RR^b^	CI 95%	RR^b^	CI 95%
Caesarean section^c^	9	(6.2)	914	(6.6)	0.839	0.92	(0.46–1.86)	0.91	(0.45–1.869)
Previous delivery	61	(42.1)	6,552	(47.5)	0.190	0.77	(0.53–1.13)	0.71	(0.48–1.03)
Multiple birth^c^	3	(2.1)	300	(2.2)	0.930	0.97	(0.31–3.08)	1.05	(0.33–2.26)
Stillborn child^c^	2	(1.4)	66	(0.5)	0.122	2.90	(0.70–11.98)	2.66	(0.63–11.30)

a) Adjusted for low educational level and immigrant status.

b) Rate ratios from logistic regression with matching on maternal year of birth as well as month and year of most recent delivery.

c) Most recent delivery.

## Discussion

We used national registries to study all suicides among Swedish women who had given birth from 1974 throughout 2009. We calculated the risk of suicide in relation to recent delivery and the effects of mental disorder and previous self-harm on suicide after delivery.

Our first finding was a weak negative association between childbirth during the preceding year and suicide. This negative association was only significant for the entire first year. The shorter time period of 42 days and six months showed similar point estimates and the lack of significances are most likely due to the smaller sample size in these groups. Hence, no obvious temporal risk difference could be concluded from our results. Previous work has shown a markedly decreased risk of suicide the first year following childbirth [1–3], which has been interpreted as a protective effect related to childbirth and the care for a new life. However, these findings may reflect biological or psychological differences between mothers and women without children [4] since comparisons in the previous studies were made between mothers and women in the general population. In the present study, we matched cases and controls on the condition of motherhood in order to avoid a possible protective effect from motherhood as such. In doing so, our results suggest that previous studies may have overestimated the protective effect of recent delivery on suicide.

Our second finding was that maternal suicide within the first year after childbirth was strongly associated with current mental disorder, in particular affective, psychotic and substance use disorders. This is in line with previous findings of mental disorders among postpartum suicides [18, 26]. A recent discharge from psychiatric hospital, regardless of diagnosis and among the population at large, is known to imply a high risk of suicide, most pronounced for women with affective disorders [27]. The postpartum period is associated with an increased risk of mental disorders [5] and since controls were matched on time since delivery this would affect both groups equally. Notably, whether the onset of mental disorder occurred before or after partus was not taken into account. The latter may affect the interpretation of a potential causal mechanism between partus, mental disorder and suicide. However, the findings high-lights the importance of mental disorder in suicide risk assessment among mothers with recent delivery. Further, a history of self-harm was strongly associated with suicide during the first year after delivery, and is a well-known risk factor in general for repeated suicidal behaviour, including completed suicide [24, 28–30].

We found a strong association between low education and suicide in the first postpartum year. This effect seemed robust and was not affected by young age or motherhood per se, since controls were age-matched mothers. Previous work has demonstrated an association between low education and attempted suicide [11] and there is also evidence of an increased risk of suicide in the general population for those with a low income and unemployment [31].

An association between stillbirth and increased numbers of suicide attempts has previously been reported [14], and in our data there was a statistically non-significant association between stillbirth and completed suicide (aRR 2.66, 95% CI 0.63–11.30). Since stillbirths are uncommon, even large samples, like the present study, may be underpowered to detect actual risk of suicide among these mothers.

### Strengths and limitations

The present study is based on national registers that hold prospectively collected data, with high coverage and validity [23]. In combination with a long follow-up period we have been able to include a large number of suicides and hence, high statistical power. Further, the design allowed us to investigate different diagnostic groups and the associations with previous self-harm. By use of maternal controls, we have eliminated a potential bias derived from differences between mothers and women without children.

Some limitations should be noted; because register data were used, the present state of the mental disorder is not known. We have tried to minimize this uncertainty by including only discharges from psychiatric care within one year of the suicide or corresponding index date for controls. In the analysis, corresponding to our first aim, time between delivery and index event varied between individuals, and the prevalence of mental disorders is affected by the proximity of a delivery. Therefore, presence of a mental disorder could not be included in the regression model. The use of register data may also have resulted in an underestimated prevalence of mental disorder. Mental disorders of subclinical nature and with rapid onset may be undetected until death by suicide and were thus not recorded in registers. Also, we refrained from an analysis of the discrepancy between previously existing mental disorders prior to delivery or new onset of such disorders.

In conclusion, we could not confirm the previously established strong association between recent delivery and reduced risk of suicide. The weak negative association was independent of socio-economic status and previous self-harm. We further conclude that general risk factors of suicide, such as a history of self-harm and current mental disorders, are applicable when making clinical assessments of the risk of suicide in the postpartum period.
